# Diversification of von Willebrand Factor A and Chitin-Binding Domains in Pif/BMSPs Among Mollusks

**DOI:** 10.1007/s00239-024-10180-1

**Published:** 2024-06-12

**Authors:** Keisuke Shimizu, Lumi Negishi, Hitoshi Kurumizaka, Michio Suzuki

**Affiliations:** 1 Research Institute for Global Change, Japan Agency for Marine-Earth Science and Technology, 2-15 Natsushima-Cho, Yokosuka, Kanagawa 237-0061 Japan; 2 Department of Applied Biological Chemistry, Graduate School of Agricultural and Life Sciences, The University of Tokyo, 1-1-1 Yayoi, Bunkyo, Tokyo, 113-8657 Japan; 3 Institute for Quantitative Biosciences, The University of Tokyo, 1-1-1 Yayoi, Bunkyo, Tokyo, 113-8657 Japan

**Keywords:** Evolution, Mollusca, Mineralization, von Willebrand factor A

## Abstract

Pif is a shell matrix protein (SMP) identified in the nacreous layer of *Pinctada fucata* (Pfu) comprised two proteins, Pif97 and Pif 80. Pif97 contains a von Willebrand factor A (VWA) and chitin-binding domains, whereas Pif80 can bind calcium carbonate crystals. The VWA domain is conserved in the SMPs of various mollusk species; however, their phylogenetic relationship remains obscure. Furthermore, although the VWA domain participates in protein–protein interactions, its role in shell formation has not been established. Accordingly, in the current study, we investigate the phylogenetic relationship between PfuPif and other VWA domain-containing proteins in major mollusk species. The shell-related proteins containing VWA domains formed a large clade (the Pif/BMSP family) and were classified into eight subfamilies with unique sequential features, expression patterns, and taxa diversity. Furthermore, a pull-down assay using recombinant proteins containing the VWA domain of PfuPif 97 revealed that the VWA domain interacts with five nacreous layer-related SMPs of *P. fucata*, including Pif 80 and nacrein. Collectively, these results suggest that the VWA domain is important in the formation of organic complexes and participates in shell mineralisation.

**Supplementary Information:**

The online version contains supplementary material available at 10.1007/s00239-024-10180-1.

## Introduction

The biominerals produced by different organisms (e.g., sponges, corals, mollusks, brachiopods, and sea urchin) are diverse in their associated minerals, microstructures, and external morphology. They comprise inorganic minerals and specific organic molecules (reviewed in Liu and Zhang 2021). The diversification and functions of these organic molecules have been investigated in myriad applied research fields, including medicine, dentistry, environmental science, and materials science.

Mollusk shells comprise calcium carbonate crystals (calcite and aragonite) and organic molecules, including polysaccharides, proteins, and lipids, that are secreted from the epithelial cells of the mantle and are incorporated into, or form scaffolds around, crystals (Belcher et al. 1996). Certain organic compounds also contribute to the formation of various shell microstructures, crystal nucleation, and crystal growth orientation (Belcher et al. 1996; Levi-Kalisman et al. 2001). The nacreous layer is lustrous and exists within the inner portion of mollusk shells, such as pearl oysters (Bivalvia), abalones (Gastropoda), and nautiluses (Cephalopoda). This characteristic laminated composite structure is formed by aragonite crystals filling compartments separated by a chitin and insoluble matrix protein framework (Gregoire 1957; Wada 1961). The organic framework may serve as a scaffold for aragonite crystal growth, with various organic molecules partially guiding the crystal growth pattern, forming a nacreous layer (Weiner and Hood 1975; Falini et al. 1996; Belcher et al. 1996).

Numerous types of matrix proteins have been identified within the nacreous layer (e.g., nacrein [Miyamoto et al. 1996], N16 [Samata et al. 1999], MSI60 [Sudo et al. 1997], and perlucin [Weiss et al. 2000]) of *Pinctada fucata*. In particular, Pif is a key protein in nacreous layer formation in the pearl oyster *P. fucata* (Suzuki et al. 2009). Pif in *P. fucata* (PfuPif) is cleaved into PfuPif 97 and PfuPif 80 at the dibasic cleavage site (RMKR); each protein with unique sequential features likely cooperates in nacreous layer formation (Suzuki et al. 2009). The cleaved proteins exhibit different features. PfuPif 97 is located within the N-terminal region of PfuPif and contains a von Willebrand factor A (VWA) domain, a chitin-binding (CB) domain, and a CB-like domain (Suzuki et al. 2009, 2013). The VWA domain is known to participate in protein–protein interactions (Tuckwell 1999; Whittaker and Hynes 2002). VWA domain-containing proteins (VWA dcps) have been reported in various metazoan skeletons, including corals (Ramos-Silva et al. 2013; Takeuchi et al. 2016b), mollusks (Zhang et al. 2012; Marie et al. 2012, 2013, 2017; Liu et al. 2015; Liao et al. 2015; Gao et al. 2015; Zhao et al. 2018; Mann et al. 2018; Shimizu et al. 2019, 2022b; Oudot et al. 2020; Takeuchi et al. 2021; Setiamarga et al. 2021; Zhang et al. 2021), and brachiopods (Luo et al. 2015). CB domain-containing proteins (CB dcps) participate in protein–chitin or other polysaccharide interactions and have been identified in shell matrix proteins (SMPs) in mollusks (Zhang et al. 2012; Marie et al. 2012, 2013, 2017; Liu et al. 2015; Liao et al. 2015; Gao et al. 2015; Zhao et al. 2018; Mann et al. 2018; Shimizu et al. 2019, 2022b; Oudot et al. 2020; Takeuchi et al. 2021; Setiamarga et al. 2021; Zhang et al. 2021). Thus, the VWA and CB domains of PfuPif 97 are thought to participate in organic framework formation in the nacreous layer (Suzuki et al. 2009).

PfuPif is cleaved into two proteins, PfuPif 97 and PfuPif 80, at the dibasic cleavage site (RMKR), and each protein with different sequential features is thought to cooperate in nacreous layer formation (Suzuki et al. 2009). The cleaved proteins exhibit different features. PfuPif 97 is the N-terminal part of PfuPif and has a von Willebrand factor A (VWA) domain, a CB domain, and a CB-like domain (Suzuki et al. 2009, 2013). The VWA domain is involved in protein–protein interactions and is composed of multiprotein complexes (Tuckwell 1999; Whittaker and Hynes 2002). VWA domain-containing proteins (VWA dcps) have been reported in various metazoan skeletons (corals: Ramos-Silva et al. 2013; Takeuchi et al. 2016b, mollusks: Zhang et al. 2012; Marie et al. 2012, 2013, 2017; Liu et al. 2015; Liao et al. 2015; Gao et al. 2015; Zhao et al. 2018; Mann et al. 2018; Shimizu et al. 2019, 2022b; Oudot et al. 2020; Takeuchi et al. 2021; Setiamarga et al. 2021; Zhang et al. 2021, brachiopod: Luo et al. 2015). CB domain-containing proteins (CB dcps) are involved in protein–chitin or other polysaccharide interactions and have also been reported as shell matrix proteins in mollusks (Zhang et al. 2012; Marie et al. 2012, 2013, 2017; Liu et al. 2015; Liao et al. 2015; Gao et al. 2015; Zhao et al. 2018; Mann et al. 2018; Shimizu et al. 2019, 2022b; Oudot et al. 2020; Takeuchi et al. 2021; Setiamarga et al. 2021; Zhang et al. 2021). Thus, PfuPif 97 has both VWA and CB domains, which are thought to participate in organic framework formation in the nacreous layer (Suzuki et al. 2009).

PfuPif 80 is the C-terminal region of the PfuPif protein, containing partial laminin G (LG) domains and low-complexity regions with numerous acidic and basic amino acid residues (Asp, Lys, and Arg) (Suzuki et al. 2009, 2013). PfuPif 80 has aragonite crystal-binding ability and contributes to aragonite formation (Suzuki et al. 2009). Similarly, BMSP (Blue Mussel Shell Protein) has common domain components (VWA, CB, and LG domains); Pif has been identified as a calcium carbonate-binding protein in the nacreous layer of the blue mussel *Mytilus galloprovincialis* (Suzuki et al. 2011). MgaBMSP is also cleaved into two proteins: MgaBMSP 120 and MgaBMSP 100. Similar to Pif 80, MgaBMSP 100 can bind to calcium carbonate crystals (Suzuki et al. 2009, 2011). However, MgaBMSP differs from PfuPif in that it contains four VWA domains in tandem (Suzuki et al. 2011). Although PfuPif and MgaBMSP were initially identified as nacreous layer-specific matrix proteins, recent omics (genomics, transcriptomics, and proteomics) studies have revealed numerous types of Pif- and BMSP-like genes or proteins in various mollusks with and without nacreous layers (Marie et al. 2012, 2017; Feng et al. 2017; Gao et al. 2015; Liao et al. 2015; Zhang et al. 2018; Takeuchi et al. 2021; Sun et al. 2020; Varney et al. 2021; Shimizu et al. 2022b; Setiamarga et al. 2021; Zhang et al. 2021). For instance, BMSP-like proteins have four VWA domains, while CB domains have been identified in the shells of bivalves *Crassostrea gigas* (Zhao et al. 2018), *Atrina pectinata* (Shimizu et al. 2022b), and *Tridacna crocea* (Takeuchi et al. 2021). Similar to MgaBMSP 100, the BMSP-like protein in *A. pectinata* exhibits a calcium carbonate-binding ability (Shimizu et al. 2022b). Furthermore, the sequence of a BMSP-like protein, containing three VWA domains, a CB domain, and a CB-like domain, was detected in the genome of the gastropod *Lotiia gigantea* (Suzuki et al. 2013) and later identified as an SMP (Marie et al. 2013).

VWA and CB domain-containing proteins (VWA–CB dcps) are diverse in lophotrochozoans, including mollusks, brachiopods, nemerteans, and phoronids (Luo et al. 2015, 2017; Zhao et al. 2018). Proteomic analyses have identified six types of VWA–CB dcps in *P. fucata*, including PfuPif, within the adult shell layers (nacreous or prismatic layers) or the D-shaped larval shell (Zhao et al. 2018). One of these in *M. galloprovincialis* (MgaBMSP) contains four VWA domains (Suzuki et al. 2011). Previous studies have suggested that various VWA–CB dcps participate in shell formation; however, each protein has a unique role in different microstructures and developmental stages. Furthermore, while many types of VWA or CB dcps have been defined as SMPs in various mollusks and designated ‘Pif-like,’ their evolutionary relationships remain unclear.

To understand the evolution of the Pif/BMSP family, in the current study, we conducted phylogenetic and functional analyses of the VWA domain. Phylogenetic analysis of VWA dcps identified a large clade designated Pif/BMSP family (Pif/BMSPf) comprising many shell-related VWA–CB dcps, including PfuPif and MgaBMSP. Pif/BMSPf was classified into eight subfamilies. We determined the spatial expression patterns of four Pif/BMSPf protein-encoding genes in the mantle tissue of *P. fucata*. Although the specific matrix proteins that interact with the VWA domains in Pif/BMSPf proteins have not been reported, we postulate that the VMA domains are key to the protein–protein interactions required for organic complex formation. Thus, we conducted pull-down assays using recombinant PfuPif proteins with altered VWA domains and SMPs extracted from the nectareous layer of *P. fucata*. Various SMPs that interact with the VWA domain of PfuPif were identified. Cumulatively, this study provides new insights into the function and evolution of Pif/BMSPf proteins in mollusks.

## Materials and Methods

### Identification of Pif/BMSP Family Proteins

VWA domain was identified from four molluscan genomes including two bivalves (*P. fucata* [Takeuchi et al. 2016a] and *C. gigas* [Zhang et al. 2012]), a gastropod *L. gigantea* [Simakov et al. 2013], a cephalopod *O. bimaculoides* [Albertin et al. 2015], and the transcriptome data of a schaphopod (*A. entalis*) (Bioproject Id: PRJNA506080) using HMMER v3.4 (*e*-values < 1.0e−5, http://hmmer.org; last accessed August 30, 2023) with protein domain database Pfam (https://pfam.xfam.org; last accessed August 30, 2023). We then selected the VWA sequences with high homology to those of PfuPif using BLSTP (*e*-value < 1.0e−10). We then conducted a molecular phylogenetic analysis using selected VWA sequences and VWA sequences from previously known SMPs or mantle-specific genes in molluscs (Suzuki et al. 2013; Liao et al. 2015; Marie et al. 2017; Zhang et al. 2018; Sun et al. 2020; Varney et al. 2021; Takeuchi et al. 2021; Shimizu et al. 2022b).

### Molecular Phylogeny

The sequences of the VWA domain regions of VWA dcps were aligned using the online version of MAFFT v7.511 (https://mafft.cbrc.jp/alignment/server/; last accessed August 30, 2023) (Kuraku et al 2013; Katoh et al. 2019). We trimmed the alignments using TrimAl (1.2rev59) (gap threshold set at 0.9) (Capella-Gutiérrez et al. 2009), and the remaining 161 residues were used for molecular phylogenetic analysis. The maximum likelihood tree was constructed with IQ-TREE v2.2.2.7 (http://www.iqtree.org/; last accessed August 30, 2023) (Minh et al. 2020) using the best-fit model (LG+G4) selected by ModelFinder (Kalyaanamoorthy et al. 2017) with 1,000 ultrafast bootstrap (UFboot) replicates and 1000 SH-aLRT branch tests.

### Identification of Specific Domains and the Partial LG Domain

We identified specific domains in Pif/BMSP family proteins using HMMER v3.4 (*e*-values < 1.0e−5, http://hmmer.org; last accessed August 30, 2023) with protein domain database Pfam (https://pfam.xfam.org; last accessed August 30, 2023). Signal peptide prediction and low complexity regions (LCR) (compositionally biased region) were conducted using SignalP (Petersen et al. 2011) and SEG (Wootton and Federhen 1996) (*e*-value < 1.0e−5) included in the online version of Simple Modular Architecture Research Tool (SMART, http://smart.embl-heidelberg.de; last accessed October 2, 2023) (Letunic et al. 2015; Letunic and Bork 2018). Some of the Pif/BMSP family proteins were known to have one or more CB domain-like sequence (Suzuki et al. 2013). Thus, we also identified the CB Peritrophin-A (CBM_14) like domain using HMMER v3.4 (http://hmmer.org; last accessed August 30, 2023) with default setting and defined two types of CBM_14-like domains (CBM_14L1 and CBM_14L2) according to their independent *e*-value (1e−5 ~ 1.0 and 1.0 ~ 10,000, respectively). To find the partial sequence of the LG domain in downstrem of the CB domain of Pif/BMSP family proteins, we used conserved sequence (N-terminal of LG domain: AYFNGRAGLKIPRFSGVPYGKSVFIKMKYKED, C-terminal of LG domain: WKTVSLKISNGHIRGRRDDREDKDVLDGDLKTTFSGFQIGQGASNKNFKGYMDEVYIYF) that have already reported in the previous study (Suzuki et al. 2013) and searched by BLASTN and the online version of MAFFT v7.511 (https://mafft.cbrc.jp/alignment/server/; last accessed August 30, 2023) (Kuraku et al 2013; Katoh et al. 2019). Pif/BMSP family proteins were classified based on the similarity in amino acid composition between the N-terminal and C-terminal sides of the LG domain using Ward’s hierarchical clustering method in R v4.3.0. Silhouette analysis was conducted using R v4.3.0, and the optimal number of clusters was determined.

### In Situ Hybridization

The mantle tissues of *P. fucata* were dissected from fresh individuals and were gifted by the Mie Prefecture Fisheries Research Institute (Mie, Japan). RNA extraction and cDNA synthesis were performed using Sepasol RNA I Super G (#09379-84, Nacalai Tesque Inc., Kyoto, Japan) and Prime Script RT reagent kit (#RR037A, Takara, Tokyo, Japan), respectively, according to the manufacturer’s protocol. Partial Pif, BMSP, Pif in nacreous layer (PifN), and complement control protein (CCP) domain-containing Pif (PifCCP) sequences from *P. fucata* were amplified via PCR using specific primers (Supplementary Table S1). 2nd PCR was conducted using T7 or SP6 tailed primers (Supplementary Table S1) and the PCR products were used for probe synthesis. Probe synthesis was conducted using DIG RNA labeling mix (#11277073910, Roche), 10 mM dithiothreitol (DTT), RNase ribonuclease inhibitor (#SIN201, Toyobo), T7 or SP6 RNA polymerase in 1X transcription buffer (#10881767001 or #10810274001, Roche), and purified PCR products (500 ng per reaction) according to the manufacturer’s protocol. RNA probes were purified using NucAway spin columns (#AM10070; Thermo Fisher Scientific). In situ hybridization was performed as previously described (Shimizu et al. 2020).

### Pull-Down Assay

The pull-down assay was performed as described previously (Shimizu et al. 2022a). The simple method is as follows: the recombinant VWA protein was prepared using the recombinant vector pET44a with the VWA sequence of PfuPif using the InFusion HD Cloning kit (#Z9648N, Takara) (Supplementary Table S1). The rVWA protein expressed in *Escherichia coli* (BL21) was purified using a Ni-column (#17531801, Ni Sepharose 6 Fast Flow, GE Healthcare, CHI, USA), and the purified proteins were confirmed by SDS–PAGE and CBB staining. Five micrograms of rVWA protein and tag-only protein (negative control) were bound to Ni-columns (#17531801, GE Healthcare), and each column was washed three times with 20 mM imidazole in wash buffer (0.5 M NaCl, 20 mM phosphate buffer pH 7.5). These columns were incubated with 50 µg of proteins were extracted from nacreous layer of *P. fucata* using acetic acid at 4ºC for 18 h. After washing five times with 20 and 50 mM imidazole in wash buffer, the bound proteins were collected using wash buffer containing 500 mM imidazole. The binding protein solutions were concentrated in a Vivaspin 500–10 K (#VS0101, Sartorius, Göttingen, Germany) and used for peptide analysis.

### LC–MS/MS Analysis

Protein alkylation was conducted with alkylation buffer (7 M Guanidine Hydrochloride, 0.5 M Tris–HCl pH 8, 10 mM EDTA pH 8) containing 5 mM DTT for 30 min at 60 ℃. After adding 2 µL of 0.5 M lodoacetamide and incubation for 1 h at 25 ℃ in dark, protein purification was performed using methanol–chloroform. The precipitation of protein was dissolved in 45 µL of trypsin solution (11 ng/µL) (Trypsin Gold [V528A, Promega, WI, USA] in 50 mM NH_4_HCO_3_ [pH 8.0]) and incubated at 37 ℃ for 18 h. After adding 5 µL of 1% TFA (final concentration is 0.1%), samples were used for peptide analysis (LC–MS/MS) (Thermo Fisher, Orbitrap Fusion Tribrid Mass Spectrometer). The data from LC–MS/MS was analyzed using the soft of Proteome Discover 2.4 and the protein database from the predicted transcripts for genome assembly ver 2.0 of *P. fucata* (pfu_aug2.0. AA.fasta) (Takeuchi et al. 2016a).

## Results

### Classification of Pif/BMSP Family Proteins in Mollusks

We identified 165, 124, 74, 111, and 32 VWA dcps in the genome and transcriptome databases of four molluscs: *P. fucata* (Bivalvia), *L. gigantea* (Gastropoda), *O. bimaculoides* (Cephalopoda), and *A. entails* (Schaphopoda), respectively (Supplementary Table S2). Within these proteins, a total of 255, 206, 94, 150, and 55 VWA domains were detected in *P. fucata*, *C. gigas*, *L. gigantea*, *O. bimaculoides*, and *A. entails*, respectively (Supplementary Table S3). To select VWA sequences for molecular phylogenetic analysis, we conducted a BLASTP homology search using the VWA domain sequence of PfuPif as a query sequence (*e*-value < 1.0e−10). We obtained 20, 31, 25, 11, and 9 (total 96) VWA sequences with high homology to the PfuPif VWA domain among the 255 (*P. fucata*), 206 (*C. gigas*), 94 (*L. gigantea*), 150 (*O. bimaculoides*), and 55 (*A. entails*) VWA sequences, respectively (Supplementary Table S4). We then conducted a molecular phylogenetic analysis using the selected 96 VWA sequences from four mollusks combined with 29 VWA sequences from previously reported SMPs or mantle-specific genes in other mollusks. Hence, a total of 125 VWA sequences were included in our molecular phylogenetic analyses.

**Fig. 1:**
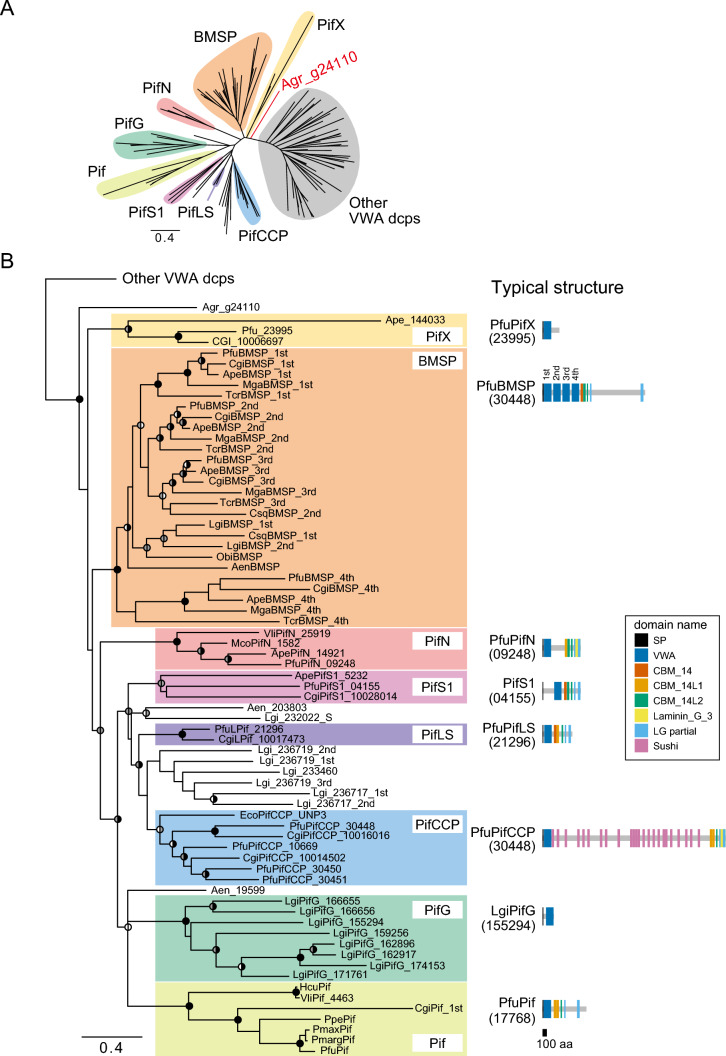
Molecular phylogeny of molluscan VWA domain-containing proteins. **A**, **B** The maximum likelihood tree was inferred from 125 VWA domain sequences using the LG+G4 model (161 positions, 1,000 ultrafast bootstrap replicates, and SH-aLRT test). The branch lengths are proportional to the expected number of substitutions per site, as indicated by the scale bar. **B** Details of the Pif/BMSP family clade (except for the VWA dcps clade) in (**A**). Schematic representation of typical domain structure of several subfamilies are shown in right side of phylogenetic tree (Schematic representation of other Pif/BMSPs are shown in Fig. 2). Black, gray, and white left half circles on nodes indicate high (≥ 95%), medium (≥ 85%), and low (≥ 75%) ultrafast bootstrap values, respectively. Black, gray, and white right half circles on nodes also indicate high (≥ 85%), medium (≥ 65%), and low (≥ 45%) SH-aLRT values, respectively. See Supplementary Table S5 for species name abbreviations

**Fig. 2:**
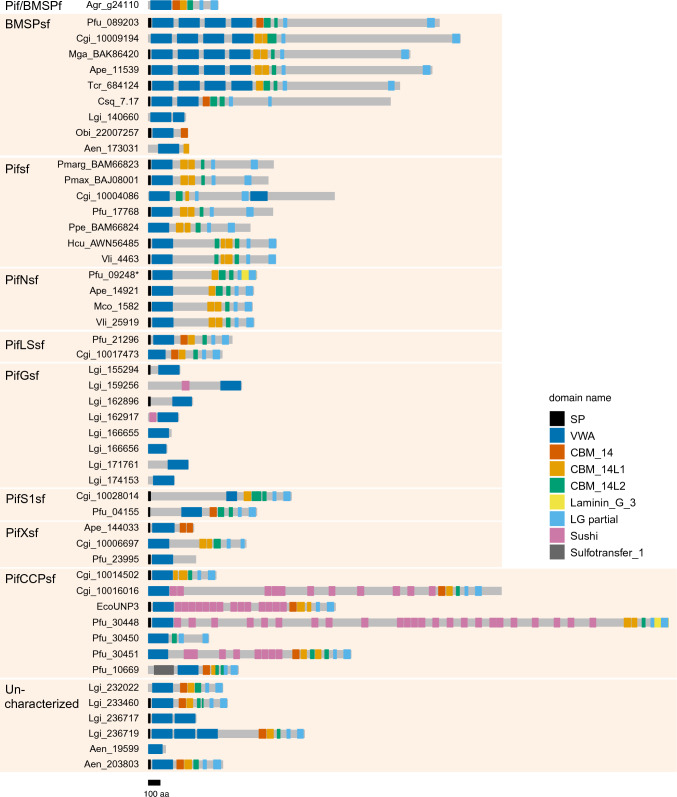
Schematic representation of VWA dcps in Pif/BMSP family. The three types of CBM_14 (CBM_14, CBM_14L1, and CBM_14L2) show differences in independent *e*-value (< 1e−5, 1.0, and 10,000, respectively) (Supplementary Table S6). *CBM_14* chitin-binding peritrophin A domain, Laminin_G_3, *LG* laminin G, *Sushi*, sushi repeat domain, *SP* signal peptide, *VWA* von Willebrand factor type A domain, *Sulfotransfer_1* sulfotransferase domain; see Supplementary Table S5 for species name abbreviations

The result showed that 70 VWA sequences in 50 VWA dcps, including PfuPif, MgaBMSP, and other Pif-like proteins, formed a clade (i.e., the Pif/BMSP family) with strong nodal support (UFBoot ≥ 95 and SH-aLRT ≥ 85) (Fig. 1, electronic supplementary material, Figure S1, Table S5). The remaining 55 VWA sequences did not contain shell-related VWA dcps. Additionally, 50 Pif/BMSP family proteins were further classified into eight subfamilies with strong (UFBoot ≥ 95 and SH-aLRT ≥ 85) or medium (UFBoot ≥ 75) nodal support (Fig. 1). Subsequently, specific domain structures were identified for these 50 Pif/BMSP members using HMMER v3.3.2 (http://hmmer.org/; last accessed August 1, 2023) (Fig. 2). Most Pif/BMSPs (38/50, 76%) contained one or more CBM_14 domains (independent *e*-value < 1e−5) and CBM_14-like domains (independent *e*-value < 1.0 [CBM_14L1] or 10,000 [CBM_14L2]) in the downstream region of the VWA domain (Fig. 2, Supplementary Table S6).

The BMSP subfamily (BMSPsf) comprises MgaBMSP and other proteins in bivalves, gastropods (*L. gigantea* and *Chrysomallon squamiferum*), cephalopods (*O. bimaculoides*), and schaphopods (*A. entalis*). All five BMSPs in bivalves (*P. fucata*, *A. pectinata*, *C. gigas*, *M. galloprovincialis*, and *Tridacna crocea*) contained four VWA domains tandemly arranged at the N-termini (Fig. 2)*,* and these VWA domains formed a single clade (Fig. 1)*.* The Pif subfamily (Pifsf) comprised PfuPif and other proteins identified as SMPs in the nacreous layer, except for the Pacific oyster *C. gigas* (CGI_10004086) (Fig. 1B). Four of the bivalve VWA dcps formed the subfamily PifN (Pif-like in the nacreous layer) as they were also identified as SMPs from the nacreous layer of *Villosa lienosa* (Marie et al. 2017), *Mytilus corsucus* (Liao et al. 2015), *A. pectinata* (Shimizu et al. 2022b), and *P. fucata* (Zhao et al. 2018) like PfuPif (Suzuki et al. 2009) (Fig. 1B). Unlike other Pif/BMSPs (except for HcuPif and VliPif), PifNsf proteins have relatively long insert sequences between the VWA and CB domains (Fig. 2). Additionally, the PifLS (Pif-like in larval shell) subfamily comprises two proteins identified as larval SMPs in *P. fucata* and *C. gigas* (Zhao et al. 2018). Meanwhile, subfamily PifG (Pif-like in gastropoda), which are found only in the gastropod *L. gigantea* contained eight VWA dcps but lacked CBM_14 and LG domains. Four of the eight PifGsf proteins had a VWA domain and CCP domains, while none were identified as SMPs of *L. gigantea* (Mann et al. 2012; Mann and Edsinger 2014). The remaining three subfamilies were only found in bivalves and had medium nodal support (UFBoot ≥ 75 and SH-aLRT ≥ 65); they were designated PifCCPsf (Pif-like with CCP domains), PifS1sf (Pif-like in shells 1), and PifXsf (Fig. 1). Members of PifCCPsf have VWA, CBM_14, and LG domains similar to other BMSP/Pif family proteins while also containing numerous Sushi/CCP domain repeats between the VWA and CB domains (Fig. 2). Two PifCCPsf proteins (Pfu_30448 and EcoUNP3) were identified as SMPs from the prismatic layer of *P. fucata* (Zhao et al. 2018) and the nacreous layer of *Elliptio complanata* (Marie et al. 2017). PifS1sf comprises three proteins identified as SMPs in *P. fucata* (larval shell)*, C. gigas* (adult shell), and *A. pectinata* (prismatic layer of adult shell) (Zhang et al. 2012; Zhao et al. 2018; Shimizu et al. 2022b). They had a relatively long insertion between the signal peptide and VWA domain, which differed from other Pif/BMSP family proteins (Fig. 2). Finally, PifXsf proteins did not have common features within this subfamily. Although the Ape_144033 protein was identified as an SMP in the nacreous layer of *A. pectinata* (Shimizu et al. 2022b), other PifXsf proteins have not been previously reported as SMPs.

### Amino Acids Compositions of the LG Domain Insert Sequences and Low-Complexity Regions

**Fig. 3:**
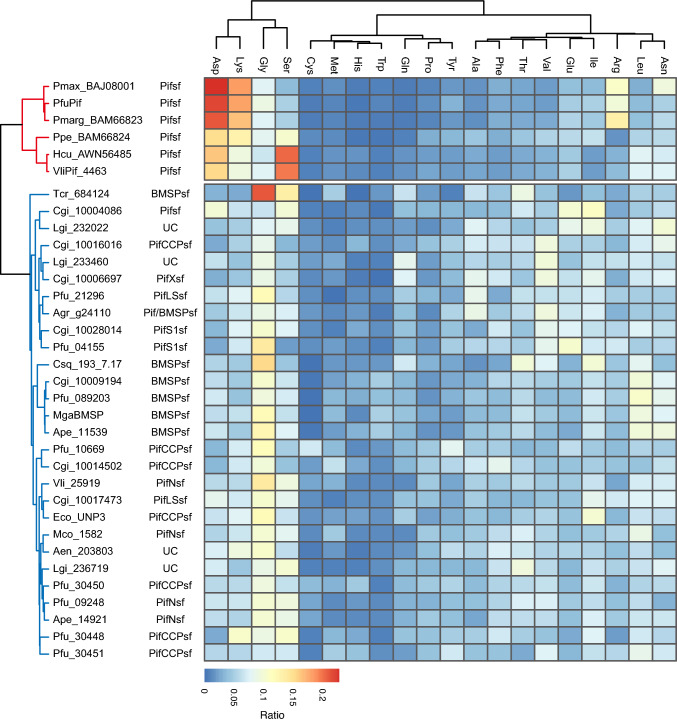
Amino acid component of insert region between partial LG domains. Heatmap shows amino acid ratio in the insert region. The result of cluster analysis shows on the left. Red and blue colored lines indicate cluster 1 (Pif subfamily proteins except for CgiPif [Cgi_10004086]) and 2 (other proteins), respectively. See Supplementary Table S5 for species name abbreviations. *UC* uncharacterized Pif/BMSP protein

Some Pif and BMSP proteins that contain partial sequences of the N-terminal and C-terminal regions of the LG domain in the downstream region of the CB domain are well-conserved (Suzuki et al. 2013). We searched for partial LG domain sequences from the 50 Pif/BMSPs identified in this study. Partial sequences of the N- and C-terminal regions of the LG domain were identified in the downstream region of the CB domain for 34 Pif/BMSPs (Fig. 2); complete sequences of the LG domain were obtained for 2/34 Pif/BMSPs (PfuPifCCP and PfuPifN) (Fig. 2). Considering that sequences of various lengths were inserted (amino acids 138–1,438), we compared the amino acid compositions of the insert sequences for 34 Pif/BMSPs. The cluster analysis results identified two clusters: six Pifsf proteins (PfuPif, PmargPif, PmaxPif, PpePif, HcuPif, and VliPif) and another 28 Pif/BMSPs (Fig. 2, Supplementary Figure S2). Furthermore, the insert regions of the Pifsf proteins were classified into two subclusters (*Pinctada* spp. and others). The insert sequence of the LG domain Pif of *Pinctada* spp. (*P. fucata*, *P. maxima*, and *P. margaritifera*) exhibited unique features with many polar amino acids (Asp, Arg, and Lys) (Fig. 3). In contrast, the Pif of freshwater pearl oysters (*H. cumingii* and *V. lienosa*) contained Ser and Asp residues in the insert region (Fig. 3). The Pif of *P. penguin* had intermediate features (Asp-, Lys-, and Ser-rich) (Fig. 3). Only CgiPif (Cgi_10004088) was separated from the Pif family proteins and classified with other Pif/BMSPs (Fig. 3).

**Fig. 4:**
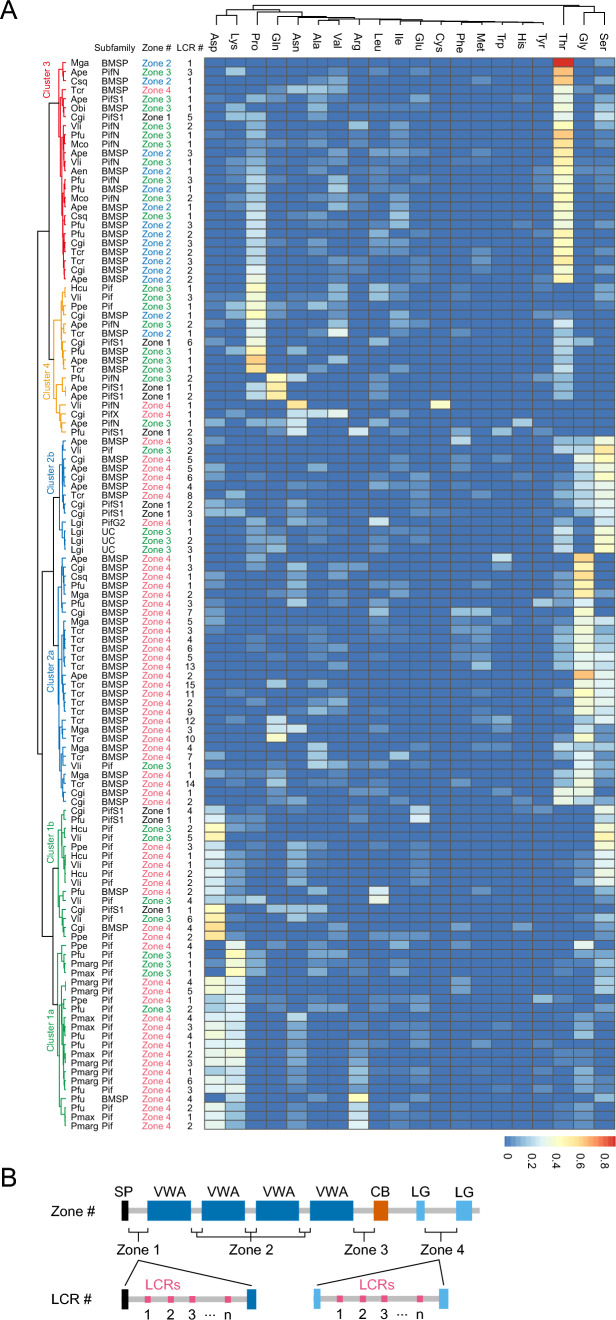
Amino acid component of low complexity regions in Pif/BMSP family proteins. **A** Heat map shows amino acid ratio in the low complexity regions. The result of cluster analysis shows on the left. See Supplementary Figure S3 for the detail of cluster analysis. Zone 1, 2, 3, and 4 indicate the upstream region of VWA domain, between tandem VWA domains, between VWA and CB domains, and between conserved LG domains, respectively (see Fig. 4B). LCR # indicates the order of LCRs in each zone (see Fig. 4B). See Supplementary Tables S5 and S7 for species name abbreviations. **B** Schematic representation of zones 1–4 in Pif/BMSP protein (upper) and the LCR # in each zone (lower). *UC* uncharacterized Pif/BMSP protein

Certain Pif/BMSPs contained one or more low-complexity regions (LCRs); 119 LCRs were detected in the 30 Pif/BMSPs in four distinct regions: zone 1, upstream of the VWA domain; zone 2, between tandem VWA domains; zone 3, VWA and CB domains; zone 4, N- and C-terminal regions of the LG domain (Fig. 4, Supplementary Table S7). The LCRs in Pifsf and BMSPsf existed almost exclusively in zone 4 (Fig. 4). In contrast, those in PifS1sf and PifNsf were primarily located in zones 1 and 3, respectively (Fig. 4). Most PifCCPsf proteins were inserted by CCP domains rather than LCRs in zone 3.

We then conducted a clustering analysis based on the amino acid composition of the 119 LCRs, generating four clusters (Fig. 4 and Supplementary Figure S3). Cluster 1 comprised LCRs in zones 3 and 4 of the Pifsf proteins and was further classified into two subclusters (Fig. 4A). Cluster 1a consisted of LCRs in zone 3 of the *Pinctada* spp. Pifsf proteins; these regions contained Asp-, Lys-, or Arg-rich sequences (Fig. 4A). In contrast, cluster 1b comprised zones 3 and 4 of Pifsf proteins in freshwater pearl oysters (*V. lienosa* and *H. cumingii*) (Fig. 4A). They also contain Asp residues. Furthermore, Ser residues were present in zone 4 of Pifsf proteins of freshwater pearl oysters (Fig. 4A). Cluster 2 was classified into two subclusters (2a and 2b), comprising LCRs in zone 4 of BMSPsf and zone 1 of PifS1sf proteins, respectively, and containing Thr residues (Fig. 4A). The former contained Gly-rich LCRs, whereas the latter contained Ser-rich LCRs (Fig. 4A). Cluster 3 primarily included LCRs in zone 2 of the BMSPsf and zone 3 of the PifNsf and contained Thr residues (Fig. 4A). Cluster 4 comprised LCRs in zone 3 of Pifsf and BMSPsf and zone 1 of PifS1. The former was characterised by Pro-rich LCRs, whereas the latter contained Gln- or Asn-rich sequences (Fig. 4A).

### Spatial Expression of Pif/BMSP Genes in the Mantle

**Fig. 5:**
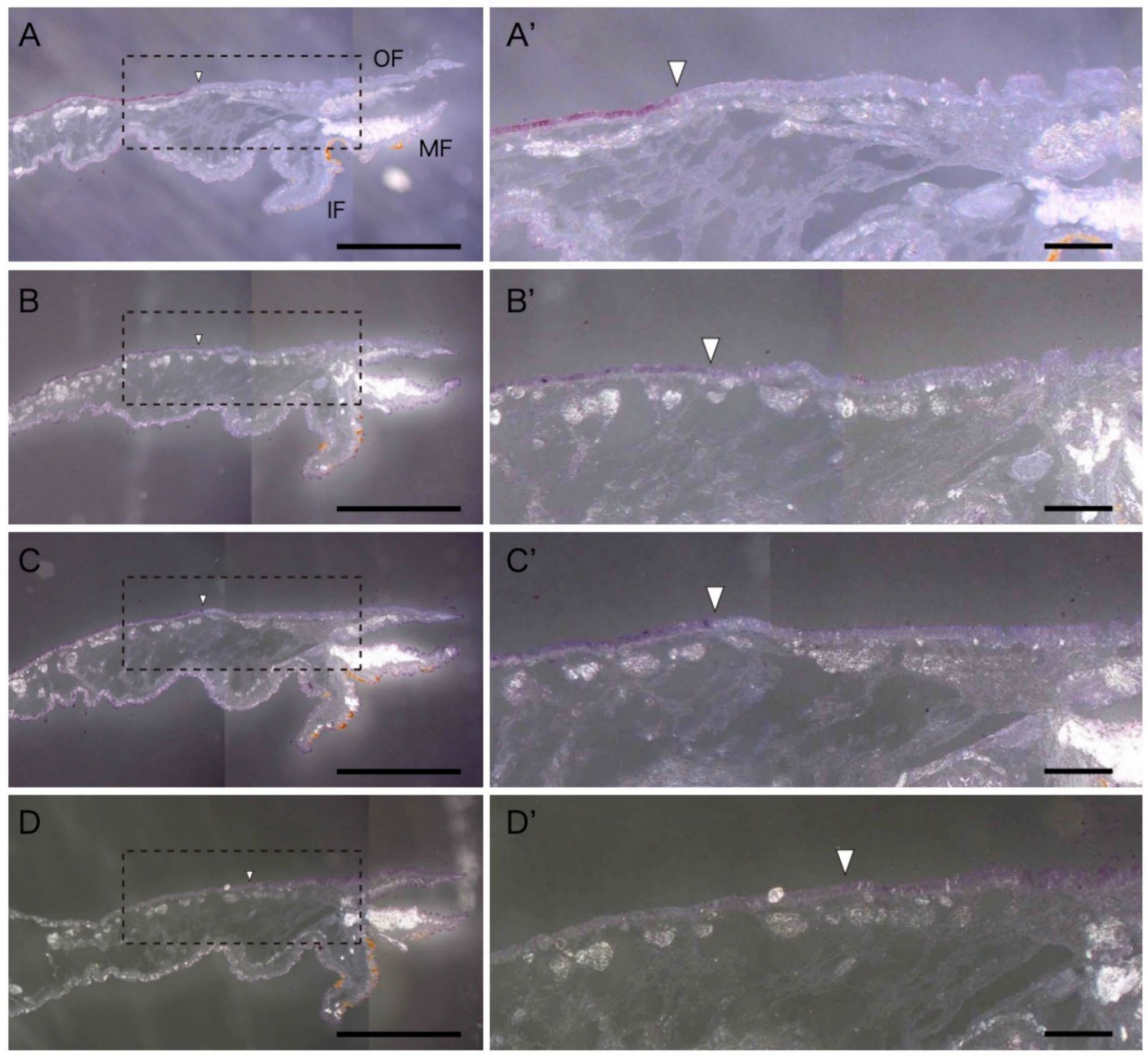
Spatial expression of shell-related Pif/BMSP genes in the mantle tissue. **A**–**D** Expression of PfuPif (**A**), PfuBMSP (**B**), PfuPifN (**C**), and PfuPifCCP (**D**) in the mantle epithelium of *P. fucata*. Positive cells are stained in purple. Upper and lower sides of pictures show dorsal and ventral sides, respectively. Scale bar, 1 mm. **A’**–**D’** Enlargement of the broken lines square in (**A**–**D**), respectively. Scale bar, 200 µm. White arrowheads indicate the boundary of gene expression. *IF* inner fold, *MF* middle fold, *MR* mantle rim, *OF* outer fold

We investigated the expression regions of four adult shell-related Pif/BMSP genes (*PfuPif, PfuBMSP*, *PfuPifN*, and *PfuPifCCP*) in *P. fucata* using in situ hybridization. They were expressed in two distinct areas of the dorsal outer epithelium of the mantle (Fig. 5). Three genes, Pfu*Pif*, Pfu*BMSP*, and Pfu*PifN,* were expressed in the mantle pallium corresponding to the nacreous layer-forming region (Fig. 5A–C). The Pfu*Pif* signal showed the highest intensity and was clearly expressed only in the mantle pallium (Fig. 5A). In contrast, signals of Pfu*BMSP* and Pfu*PifN* were detected in the mantle pallium and the inner surface of the outer fold, located near the periostracal groove, and on the surface epithelium of the middle and outer folds. The expression of Pfu*PifCCP* was relatively weak and ambiguous compared with Pfu*Pif*, Pfu*BMSP*, and Pfu*PifN* (Fig. 5D). Pfu*PifCCP* was expressed at the mantle edge corresponding to the prismatic layer-forming region (Fig. 5D).

### Protein Interaction Between the VWA Domain and Shell Matrix Proteins

To investigate the function of the Pif VWA domain, we prepared a recombinant PfuPifVWA protein and conducted a pull-down assay. We inserted the VWA domain sequence of PfuPif into the pET-44(+) vector and purified the recombinant protein (r-PifVWA) using a Ni column (Supplementary Figure S4). Pull-down assays were conducted using r-PifVWA; proteins were extracted from the nacreous layer of *P. fucata* using acetic acid. Four SMPs, namely, PfuPif 80 (C-terminal region of Pif), nacrein, and serine proteinase inhibitors (SPIs) (pfu_aug2.0_1101.1_04821. t1 and pfu_aug2.0_283.1_10559.t1) were identified as the major members of the rPifVWA-binding SMPs (more than two unique peptides) in the nacreous layer (Table 1, Supplementary Table S8).

**Table 1:** The lists of the SMPs that interacted with rPfuPifVWA

Gene_id	Protein name	Layers	Domains	Function	Reference
pfu_aug2.0_715.1_17768	Pif 80	N	LCR	Calcium binding	Suzuki et al. (2009)
pfu_aug2.0_214.1_13802	nacrein	N, S	CA	HCO_3_^−^ production, Calcium binding	Miyamoto et al. (1996), Liu et al. (2015), Zhao et al. (2018)
pfu_aug2.0_1101.1_04821	SPI	N, S	Kunitz_BPTI	Proteinase inhibition	Zhao et al. (2018)
pfu_aug2.0_283.1_10559	Kazal type SPI	N, S	Kazal	Proteinase inhibition	Zhao et al. (2018)
pfu_aug2.0_2218.1_28718	Collagen-like	N	Collagen	Framework interaction	Zhao et al. (2018)

## Discussion

### Classification of Pif/BMSPs

Both VWA and CB dcps are unique proteins in mollusks and various lophotrochozoans (Nemertea, Phoronida, and Brachiopoda) (Zhao et al. 2018; Luo et al. 2015, 2017). These proteins may expand within each phylum, resulting in domain and motif shuffling (Suzuki et al. 2013; Kocot et al. 2016). Although many Pif/BMSP-like genes or proteins containing VWA and CB domains have been reported in mollusks, their evolutionary relationships remain unclear. Our molecular phylogenetic analysis of VWA dcps in molluscs showed that shell-related Pif/BMSP families, including PfuPif and MgaBMSP, formed a clade with strong nodal support in the conchifera (Bivalvia, Gastropoda, Cephalopoda, and Scaphopoda; Fig. 1). This suggests that domain shuffling occurred in the last common ancestor of mollusks and that Pif/BMSP family members are diverse among several taxa.

BMSP is a subclade of the Pif/BMSP family and is well-conserved in the major conchifera (Bivalvia, Gastropoda, Cephalopoda, and Scaphopoda). BMSPs were first identified in the nacreous layer of the blue mussel *M. galloprovincialis* (Suzuki et al. 2011) and are primarily characterised by four tandem VWA domains. In the gastropod *L. gigantea*, the BMSP-like (Lgi236719) has two tandem VWA domains and has been detected in the shell (Marie et al. 2013; Mann and Edsinger 2014). Meanwhile, our results show that Lgi236719 did not form a clade with other BMSP subfamily members (Fig. 1). Rather, Lgi140660 was identified within the BMSP clade and also contained two tandem VWA domains (Figs. 1 and 2). In addition, the four VWA domains of bivalve BMSPs formed each clade while differing from the two gastropod VWA domains (Fig. 1). In cephalopods (*O. bimaculoides*) and schaphopods (*A. entalis*), the BMSPs contained a single VWA domain (Figs. 1 and 2). These results suggest that the ancestral BMSP contained one VWA domain, the duplication of which occurred independently in the leading bivalves and gastropods.

BMSP expression in *P. fucata* was observed in the dorsal region of the outer epithelium of the mantle pallium, corresponding to the nacreous layer-forming region (Fig. 5). The expression of BMSPs in the gastropod *C. squamiferum* (Csq193_7.17) has also been reported specifically in mantle epithelial cells (Sun et al. 2020), similar to bivalve BMSPs (Fig. 5). Thus, the ancestral BMSP in conchifera might have been expressed in mantle epithelial cells and participated in the nacreous layer as well as in the formation of various shell microstructures, such as crossed lamellar.

Pif is a well-known SMP found in the nacreous layers of *P. fucata* (Suzuki et al. 2009)*.* Many Pif-like proteins containing VWA and CB domains have been identified as SMPs (or mantle-specific genes) in bivalves and other mollusks (gastropods, cephalopods, and polyplacophora) (Sun et al. 2020). However, molecular phylogenetic analysis has shown that Pifsf is a unique Pteriomorphia (Suzuki et al. 2009, 2013; Marie et al. 2012) and Unionida subfamily, including *V. lienosa* (Marie et al. 2017) and *H. cumingii* (Zhang et al. 2018). Other Pif-like proteins belonging to the Pif/BMSP family that are not homologues of PfuPif have been previously annotated (Fig. 1). All Pif homologues have been identified in the nacreous layer (Suzuki et al. 2009; Marie et al. 2012, 2017; Zhang et al. 2018), with the PfuPif-encoding gene highly expressed in the dorsal region of the outer epithelium of the mantle pallium, corresponding to the nacreous layer-forming region (Fig. 5).

We annotated a new nacreous layer-related Pif protein (i.e., PifN) that is an SMP found in the nacreous layer of four bivalves: *P. fucata* (Zhao et al. 2018), *A. pectinate* (Shimizu et al. 2022b), *M. coruscus* (Liao et al. 2015), and *V. lienosa* (Marie et al. 2017). They have relatively long insert sequences, including the poly-threonine sequence between the VWA and CB domains, compared to other Pif/BMSP family proteins, excluding PifCCP (Figs. 2, 4). Most Pif/BMSP family members have partial LG domain sequences in the C-terminus, whereas PfuPifN has a complete sequence (Fig. 2). Hence, the ancestral features of Pif/BMSP family proteins may remain in PfuPifN, with a unique sequence, including low-complexity regions, inserted.

Most Pif/BMSP family members comprise only VWA, CB, and LG domains. However, PifCCP contains many repeating shushi/CCP domains between the VWA and CB domains (Fig. 2) (Marie et al. 2017; Zhao et al. 2018). Unlike the PfuPif, PfuPifN, and PfuBMSP proteins that have been identified in the nacreous layer, PfuPifCCP is an SMP that exists in the prismatic layer of *P. fucata* (Zhao et al. 2018). Its expression pattern also differs from that of Pfu*Pif*, Pfu*PifN*, and Pfu*BMSP*; that is, Pfu*PifCCP* is expressed in the mantle edge corresponding to the prismatic layer-forming region rather than the mantle pallium corresponding to the nacreous layer-forming region (Fig. 5). This spatial expression pattern is consistent with previous transcriptome analysis results (Zhao et al. 2018). In other pearl oysters, *P. margaritifera*, PUSP11 and PUSP12 comprise two and four Sushi/CCP repeats without VWA, CB, or LG domains, respectively, and are located within the prismatic layer (Marie et al. 2012). Given the incomplete assembly of the gene database, these proteins may represent partial sequences of PifCCP proteins in *P. margaritifera*. Other Sushi/CCP domain-containing proteins have also been reported as SMPs in mollusks (Marie et al. 2012, 2013, 2017; Mann and Edsinger 2014; Mann et al. 2018; Shimizu et al. 2019) and brachiopods (Jackson et al. 2015; Luo et al. 2015). Although Sushi/CCP repeats may be related to shell formation, their function in biomineralization remains unknown. Nevertheless, with the exception of PifCCP, the combination of the Sushi/CCP domain with the VWA, CB, and LG domains has not been previously reported as an SMP in mollusks or brachiopods.

Considering that PifLS1 proteins have been identified in the larval shells of *P. fucata* and *C. gigas*, they may be involved in larval shell formation in bivalves (Zhao et al. 2018). However, it remains unclear how these proteins are employed during larval shell formation. In addition, homologs of these larval Pif proteins were not identified in a gastropod genome database for *L. gigantea*. Although proteomic analysis of the gastropod larval shells has not yet been reported, this result suggests that PifLS1sf evolved in bivalves (or pteriomorph bivalves). Indeed, the results of the molecular phylogenetic analysis showed that only BMSPsf were present in the major conchifera (Bivalvia, Gastropoda, Cephalopoda, and Scaphopoda), while members of other subfamilies were likely diverse across each class (Bivalvia and Gastropodal; Fig. 1). One Pif/BMSP subfamily, PifG1, and one unknown clade were detected in the gastropod *L. gigantea*, one of which contained SMPs (Lgi_236719 and Lgi_232022) identified in the adult shells of this species (Mann et al. 2012; Mann and Edsinger 2014).

### Possible Function of Pif/BMSP Family Proteins in Shell Formation

Various extracellular matrix proteins have been identified in biominerals, including bones and teeth in vertebrates and external skeletons in invertebrates. Most VWA dcps are components of the extracellular matrix (Tuckwell 1999). However, although they are commonly found in biominerals as skeleton matrix proteins (Liu and Zhang 2021), their functions in biomineralization remain unclear. The Von Willebrand factor (VWF) mediates platelet adhesion to collagen (Tuckwell 1999). In general, VWA DCPs, such as VWF, are involved in cell adhesion and multiprotein complex formation (Tuckwell 1999; Whittaker and Hynes 2002). Thus, most previous studies on biomineralization have hypothesised that VWA dcps participate in the protein–protein interactions among other matrix proteins. However, matrix proteins that interact with the VWA domain of biomineral proteins to form organic complexes have never been reported.

Using a pull-down assay of rPfuPifVWA, we revealed that the VWA domain region of PfuPif specifically interacts with four previously known SMPs (Pif 80, nacrein, and two serine proteinase inhibitors) (Supplementary Table S8). PfuPif is cleaved into PfuPif 97 and PfuPif 80 (Suzuki et al. 2009). PfuPif 97 consists of VWA and CB domains that can interact with chitin, whereas PfuPif 80 has an aragonite-binding ability (Suzuki et al. 2009). Furthermore, the results of gel filtration high-performance liquid chromatography (HPLC) and SDS–PAGE analyses showed that PfuPif 97, PfuPif 80, and PfuN16—the major proteins in the nacreous layer (Samata et al. 1999)—may form a high-molecular–weight complex (Suzuki et al. 2009). In addition, Suzuki et al. (2009) hypothesised that PfuPif 97 and PfuPif 80 form a complex, resulting in interactions with chitin (an organic sheet) and the nucleation of aragonite crystals. Our pull-down assay results support these findings; PfuPif 97 can bind to PfuPif 80 via the VWA domain region and likely forms a complex. In contrast, the interaction between PfuN16 and PfuPif 97 or PfuPif 80 was likely not mediated through the VWA domain.

We also identified Pfunacrein as a protein that complexes with PfuPif 97, in addition to PfuPif 80. Nacrein is a shell-related carbonic anhydrase (CA) in the nacreous and prismatic layers of *P. fucata* (Miyamoto et al. 1996; Miyashita et al. 2002). This enzyme catalyses the hydration of carbon dioxide and provides bicarbonate ions. Nacrein contains a specific Gly–Xaa–Asn repeat (Xaa = Asp, Asn, or Glu) between the CA subdomains, which interacts with calcium ions and participates in calcium carbonate crystal formation (Miyamoto et al. 1996). However, nacrein is an acid- or EDTA-soluble protein that may not readily interact directly with the insoluble organic matrix sheets composed of chitin fibrils, which form the basis of aragonite crystal growth in the nacreous layer. Therefore, PfuPif 97 with VWA and CB domains can bridge the nacrein and the organic matrix sheets to efficiently promote the nucleation and growth of calcium carbonate crystals.

Finally, rPfuPifVWA also interacted with two types of SPIs, Kunitz-type (pfu_aug2.0_1101.1_04821) and Kazal-type (pfu_aug2.0_283.1_10559) (Table 1). Many Kunitz-type SPIs have been identified within the nacreous or prismatic layers of pearl oysters, *P. fucata*, *P. margaritifera*, *P. maxima* (Marie et al. 2012; Liu et al. 2015; Zhao et al. 2018), and other bivalves (Gao et al. 2015; Liao et al. 2015, 2019; Marie et al. 2017; Shimizu et al. 2022b) and were clustered in the genome of *P. fucata* (Takeuchi et al. 2016a). Although the function of SPIs in shell formation is unclear, their diversification likely indicates their involvement in biomineralisation (e.g., regulation of matrix maturation) (Arivalagan et al. 2017; Dombre et al. 2016). The zona pellucida (ZP) domain is also involved in extracellular protein–protein interactions, such as the VWA domain (Jovine et al. 2002, 2005, 2006). Various ZP domain-containing proteins have been identified from skeletal matrix proteins in corals (Ramos-Silva et al. 2013; Takeuchi et al. 2016b) and gastropods (Marie et al. 2013; Mann and Edsinger 2014), while a portion of the ZP domain has also been identified as SMPs in bivalves (Shimizu et al. 2022a). The results of the pull-down assay using recombinant ZP protein and SMPs extracted from the nacreous layer of *P. fucata* showed that the ZP protein interacts with other SMPs, such as proteinase inhibitors (SPIs) and BMSP 100 (Shimizu et al. 2022a). However, the SMPs interacting with the ZP domain (Shimizu et al. 2022a) and the VWA domain (this study) differ slightly. These results suggest that different types of organic complexes are formed in the shell-forming region by different protein mediators (e.g., VWA dcps and ZP dcp) with roles in the formation of various microstructures.

### Evolution of Pif/BMSP Family Members in Mollusks

In the pearl oyster, *P. fucata*, 17 types of VWA–CB dcps were found in the genome database, six of which were identified in adult and larval shells (Zhao et al. 2018). Moreover, a BLAST search revealed that the VWA domains of nine VWA–CB dcps exhibited similarity with the VWA of PfuPif (*e*-value < 1.0e−10). Thus, the remaining seven VWA–CB dcps likely differed from the ‘Pif/BMSP family’ (Supplementary Figure S5). Phylogenetic analysis of VWA domains further showed that six shell-related VWA–CB dcps and three other VWA–CB dcps (total nine) formed a ‘Pif/BMSP family’ clade. Thus, the origin of the VWA domain in the Pif/BMSP family differs from that in other VWA–CB dcps. Hence, VWA–CB dcps likely evolved at least twice independently by domain shuffling. Pif/BMSP family members have evolved in the common Mollusca ancestor and are diverse in several taxa. This novel protein likely participates in shell formation as most Pif/BMSP family genes are expressed in the mantle tissue, and some have been identified as SMPs (Suzuki et al. 2009, 2013; Zhang et al. 2012; Marie et al. 2013, 2017; Mann and Edsinger 2014; Liao et al. 2015; Gao et al. 2015; Takeuchi et al. 2021; Shimizu et al. 2022b). Ancestral molluscs are thought to have mineralised structures such as shell(s) and sclerites (Scherholz et al. 2013; Vinther et al. 2017; Giribet and Edgecombe 2020), which are composed of organic matrices, such as extracellular matrix proteins, and polysaccharides, such as chitin. A previous genome study of a chiton *A. granulata* (Varney et al. 2021) showed that a VWA–CB dcp (Agr_g24110) called Pif-like (Varney et al. 2021) was highly expressed in the girdle, which involves sclerites formation compared with other tissues. The phylogenetic analysis further revealed that Agr_g24110 is located in the base of Pif/BMSP family members in mollusks (Fig. 1). Thus, the common ancestor of mollusks contains Pif/BMSP, which may also be involved in the formation of mineralised structures (shells and sclerites).

Aside from bivalves, BMSPs have also been identified in other conchifera (Gastropoda, Cephalopoda, and Schaphopoda) (Fig. 1). This suggests that BMSPs are ancestral Pif/BMSPs in the common conchifera ancestor and are involved in shell formation. Various microstructures (e.g., crossed lamellar, prismatic, and nacreous layers) have been observed in the shells of several conchifera (Marin et al. 2012), with diverse Pif/BMSPs in several groups (Fig. 1). In fact, six Pif/BMSP-encoding genes in *P. fucata* were expressed in different parts of the mantle tissue (mantle edge or mantle pallium; Fig. 5) at different developmental stages (D-shaped larva or adult) and within the shells of adults and larva (Zhao et al. 2018).

PfuPif was first identified as a nacreous layer-specific matrix protein in the pearl oyster *P. fucata* (Suzuki et al. 2009) and is specifically expressed in the epithelial cells of the mantle pallium (Fig. 5). Nacre is commonly observed in other bivalves (paleotaxodonts, pteriomorphids, paleoheterodonts, and anomalodesmata) and has evolved independently (Carter 1990). However, homologues of Pif proteins were identified as SMPs in the nacreous layers of the freshwater mussels *V. lienosa* and *H. cumingii* (Marie et al. 2017; Zhang et al. 2018) (Fig. 1). The C-terminal region of Pif/BMSPs contains two conserved regions of the LG domain (Suzuki et al. 2013). The insertion sequences in the Pif subfamily differ from those in other subfamilies (Fig. 3). Although the domain structures of Pif/BMSPs are similar (Fig. 2), the insertion sequences, including many low-complexity regions between the conserved partial sequences of the LG domain in the Pifsf, are quite different from those of other subfamilies (Figs. 3, 4). Furthermore, they differed slightly between the genus *Pinctada* and the order Unionida. Many basic residues, Lys (K) and Arg (R), exist in *Pinctada* spp. (17–19% and 10–15%, respectively) but are relatively minor in Unionida (around 8% and 2–4%, respectively). Instead of basic residues, many Ser (S) residues exist in Unionida (25–26%), not *Pinctada* spp. (3–4%; Fig. 3). In contrast, the acidic residue Asp (D) is the major residue in the *Pinctada* and Unionida (24–26% and 21–23%, respectively; Fig. 3); hence, the Asp-rich sequence is likely important for the interaction with calcium ions and shell mineralisation. Meanwhile, the insert sequence in *P. penguin* was intermediate between those of *Pinctada* and Unionida (Fig. 3). This suggests that Pif 80 and Pifs in other Pinctada species (*P. margaritifera* and *P. maxima*) evolved by accumulating mutations and through natural selection in each lineage. Moreover, these proteins were likely involved in independently evolved well-ordered nacreous layer formation.

## Conclusion

Various microstructures exist within the molluscan shells; their formation is precisely regulated by secretions of organic matrix components from the mantle epithelial cells. In the current study, we focused on PfuPif—a well-known SMP in the nacreous layer of *P. fucata—*revealing a portion of the function and evolution of Pif/BMSP family members in mollusks. PfuPif is cleaved to form PfuPif 97 and PfuPif 80. The former comprises VWA and CB domains that are highly conserved in other mollusk SMPs. We identified SMPs that interact with the VWA domain of PfuPif 97*,* including PfuPif 80, which binds to calcium carbonate crystals. Our findings support the hypothesis that PfuPif 97 and PfuPif 80 form a complex, resulting in interactions with chitin (an organic sheet) and the nucleation of aragonite crystals (Suzuki et al. 2009).

We also conducted a molecular phylogenetic analysis using VWA domain sequences because Pif/BMSPf proteins that contain VWA and CB domains were detected within the nacreous layer and other microstructures; however, their molecular evolution is unclear. We found that Pif/BMSPf proteins were classified into eight subfamilies (BMSPsf, Pifsf, PifNsf, PifLSsf, PifGsf, PifS1sf, PifXsf, and PifCCPsf) that exhibited different sequential features, expression patterns, and diversity in different taxa. Furthermore, Pif/BMSPf proteins contained one or more LCRs with amino acid components and insert regions that differed among subfamilies. Our results showed that Pif proteins in pearl oysters (genus *Pinctada*) and freshwater pearl oysters (Unionida) have independently evolved more characteristic LCRs (D, S, K, or R-rich) than other Pif/BMSP family members; these features probably correlate with well-ordered nacreous layer formation. Collectively, the findings of this study provide useful insights regarding the molecular mechanisms of shell mineralisation as well as applied research using biomimetic mineralisation in medicine, dentistry, or materials science.

### Supplementary Information

Below is the link to the electronic supplementary material.

Supplementary file1 (PDF 7330 KB)

Supplementary file2 (XLSX 90 KB)
