# Supplementary material for: Diversification of von Willebrand Factor A and Chitin-Binding Domains in Pif/BMSPs Among Mollusks
Source: J Mol Evol. 2024 Jun 12;92(4):415–31. doi: 10.1007/s00239-024-10180-1 (PMC11291548; doi:10.1007/s00239-024-10180-1)
Supplement: Supplementary file 1 — Supplementary file1 (PDF 7330 KB) [file 239_2024_10180_MOESM1_ESM.pdf]

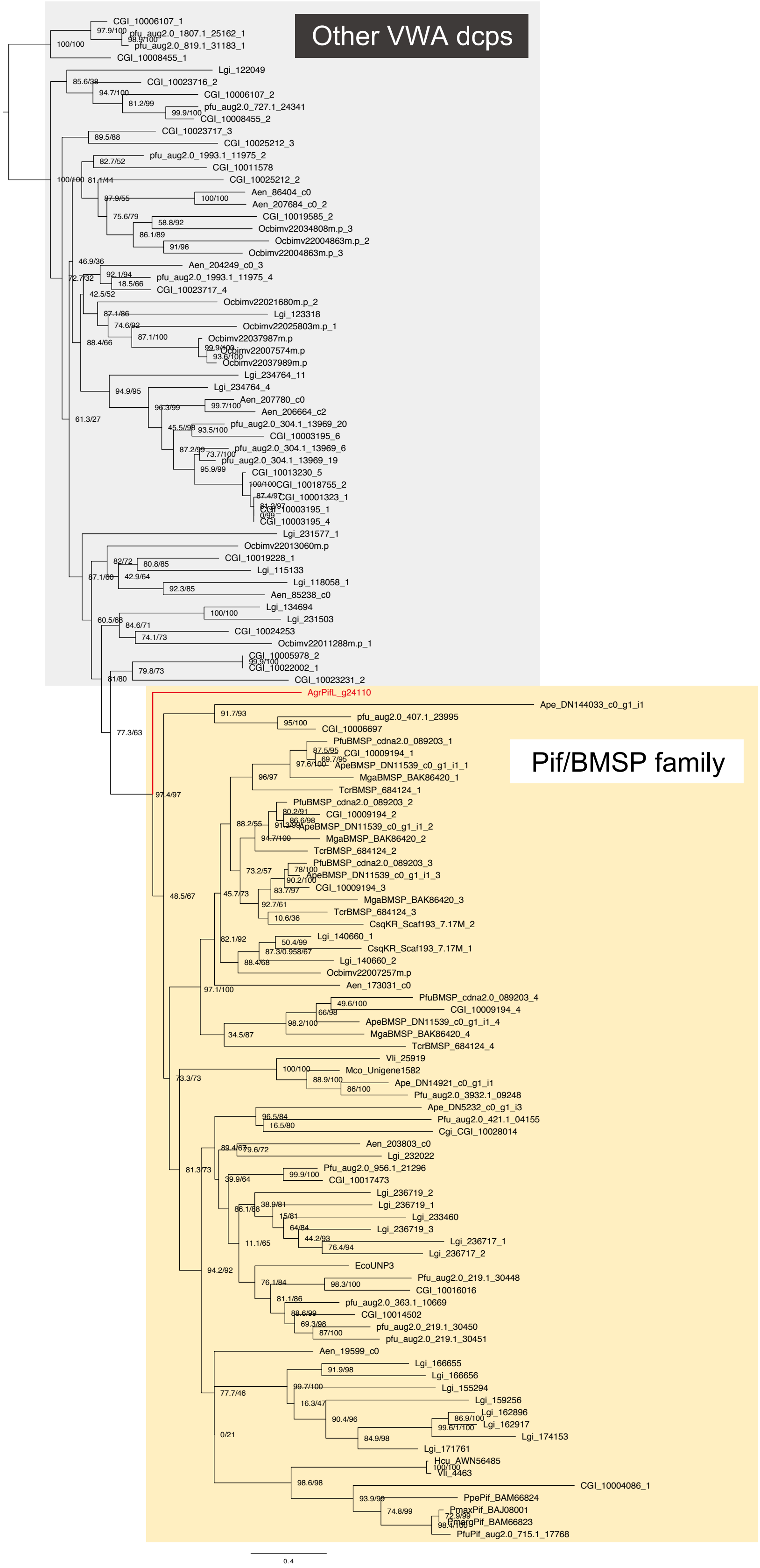


**Supplementary Figure S1.** Molecular phylogeny of VWA dcps in mollusks. Detail of phylogenetic tree in Figure 1.

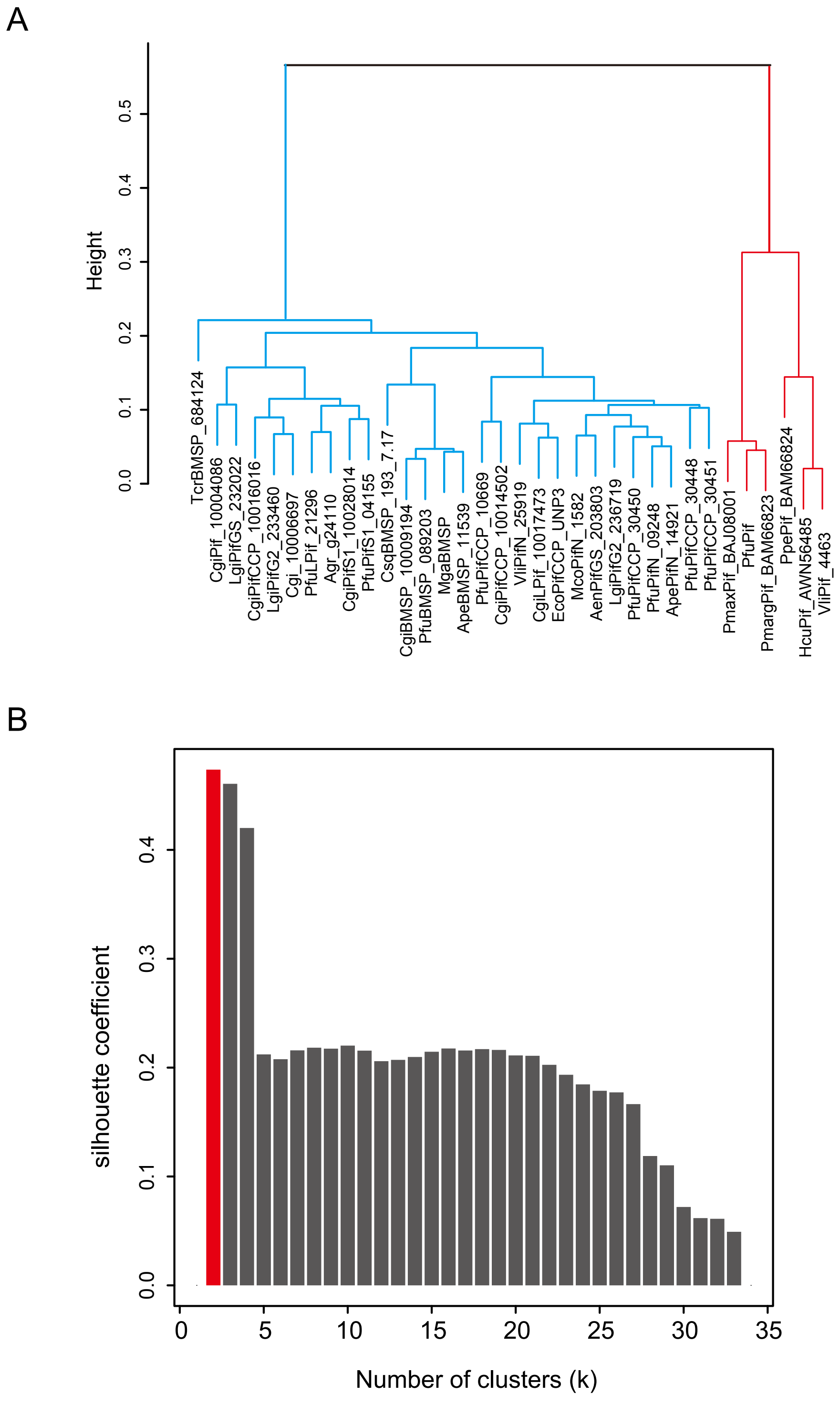


**Supplementary Figure S2. A** Cluster analysis of the amino acid compositions of insert sequences between LG domains. Red and blue lines indicate cluster 1 and 2, respectively. **B** The result of Silhouette analysis. Optimal number of clusters was estimated to two (red bar).

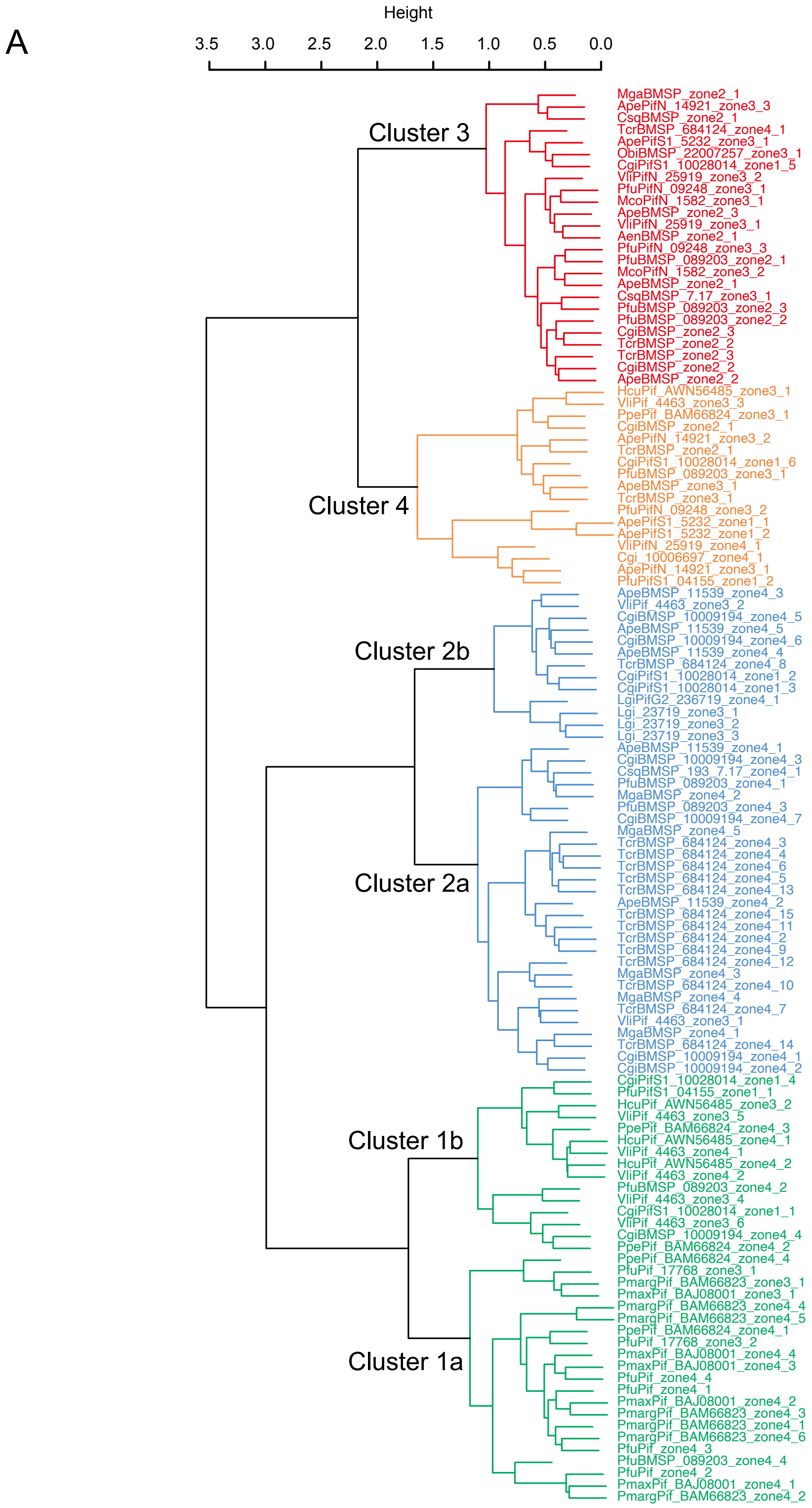
A
Height
3.5
3.0
2.5
2.0
1.5
1.0
0.5
0.0
Cluster 3
Cluster 4
Cluster 2b
Cluster 2a
Cluster 1b
Cluster 1a
MgaBMSP_zone2_1
ApePifN_14921_zone3_3
CsqBMSP_zone2_1
TcrBMSP_684124_zone4_1
ApePifS1_5232_zone3_1
ObiBMSP_22007257_zone3_1
CgiPifS1_10028014_zone1_5
VliPifN_25919_zone3_2
PfuPifN_09248_zone3_1
McoPifN_1582_zone3_1
ApeBMSP_zone2_3
VliPifN_25919_zone3_1
AenBMSP_zone2_1
PfuPifN_09248_zone3_3
PfuBMSP_089203_zone2_1
McoPifN_1582_zone3_2
ApeBMSP_zone2_1
CsqBMSP_7.17_zone3_1
PfuBMSP_089203_zone2_3
PfuBMSP_089203_zone2_2
CgiBMSP_zone2_3
TcrBMSP_zone2_2
TcrBMSP_zone2_3
CgiBMSP_zone2_2
ApeBMSP_zone2_2
HcuPif_AWN56485_zone3_1
VliPif_4463_zone3_3
PpePif_BAM66824_zone3_1
CgiBMSP_zone2_1
ApePifN_14921_zone3_2
TcrBMSP_zone2_1
CgiPifS1_10028014_zone1_6
PfuBMSP_089203_zone3_1
ApeBMSP_zone3_1
TcrBMSP_zone3_1
PfuPifN_09248_zone3_2
ApePifS1_5232_zone1_1
ApePifS1_5232_zone1_2
VliPifN_25919_zone4_1
Cgi_10006697_zone4_1
ApePifN_14921_zone3_1
PfuPifS1_04155_zone1_2
ApeBMSP_11539_zone4_3
VliPif_4463_zone3_2
CgiBMSP_10009194_zone4_5
ApeBMSP_11539_zone4_5
CgiBMSP_10009194_zone4_6
ApeBMSP_11539_zone4_4
TcrBMSP_684124_zone4_8
CgiPifS1_10028014_zone1_2
CgiPifS1_10028014_zone1_3
LgiPifG2_236719_zone4_1
Lgi_23719_zone3_1
Lgi_23719_zone3_2
Lgi_23719_zone3_3
ApeBMSP_11539_zone4_1
CgiBMSP_10009194_zone4_3
CsqBMSP_193_7.17_zone4_1
PfuBMSP_089203_zone4_1
MgaBMSP_zone4_2
PfuBMSP_089203_zone4_3
CgiBMSP_10009194_zone4_7
MgaBMSP_zone4_5
TcrBMSP_684124_zone4_3
TcrBMSP_684124_zone4_4
TcrBMSP_684124_zone4_6
TcrBMSP_684124_zone4_5
TcrBMSP_684124_zone4_13
ApeBMSP_11539_zone4_2
TcrBMSP_684124_zone4_15
TcrBMSP_684124_zone4_11
TcrBMSP_684124_zone4_2
TcrBMSP_684124_zone4_9
TcrBMSP_684124_zone4_12
MgaBMSP_zone4_3
TcrBMSP_684124_zone4_10
MgaBMSP_zone4_4
TcrBMSP_684124_zone4_7
VliPif_4463_zone3_1
MgaBMSP_zone4_1
TcrBMSP_684124_zone4_14
CgiBMSP_10009194_zone4_1
CgiBMSP_10009194_zone4_2
CgiPifS1_10028014_zone1_4
PfuPifS1_04155_zone1_1
HcuPif_AWN56485_zone3_2
VliPif_4463_zone3_5
PpePif_BAM66824_zone4_3
HcuPif_AWN56485_zone4_1
VliPif_4463_zone4_1
HcuPif_AWN56485_zone4_2
VliPif_4463_zone4_2
PfuBMSP_089203_zone4_2
VliPif_4463_zone3_4
CgiPifS1_10028014_zone1_1
VliPif_4463_zone3_6
CgiBMSP_10009194_zone4_4
PpePif_BAM66824_zone4_2
PpePif_BAM66824_zone4_4
PfuPif_17768_zone3_1
PmargPif_BAM66823_zone3_1
PmaxPif_BAJ08001_zone3_1
PmargPif_BAM66823_zone4_4
PmargPif_BAM66823_zone4_5
PpePif_BAM66824_zone4_1
PfuPif_17768_zone3_2
PmaxPif_BAJ08001_zone4_4
PmaxPif_BAJ08001_zone4_3
PfuPif_zone4_4
PfuPif_zone4_1
PmaxPif_BAJ08001_zone4_2
PmargPif_BAM66823_zone4_3
PmargPif_BAM66823_zone4_1
PmargPif_BAM66823_zone4_6
PfuPif_zone4_3
PfuBMSP_089203_zone4_4
PfuPif_zone4_2
PmaxPif_BAJ08001_zone4_1
PmargPif_BAM66823_zone4_2

B

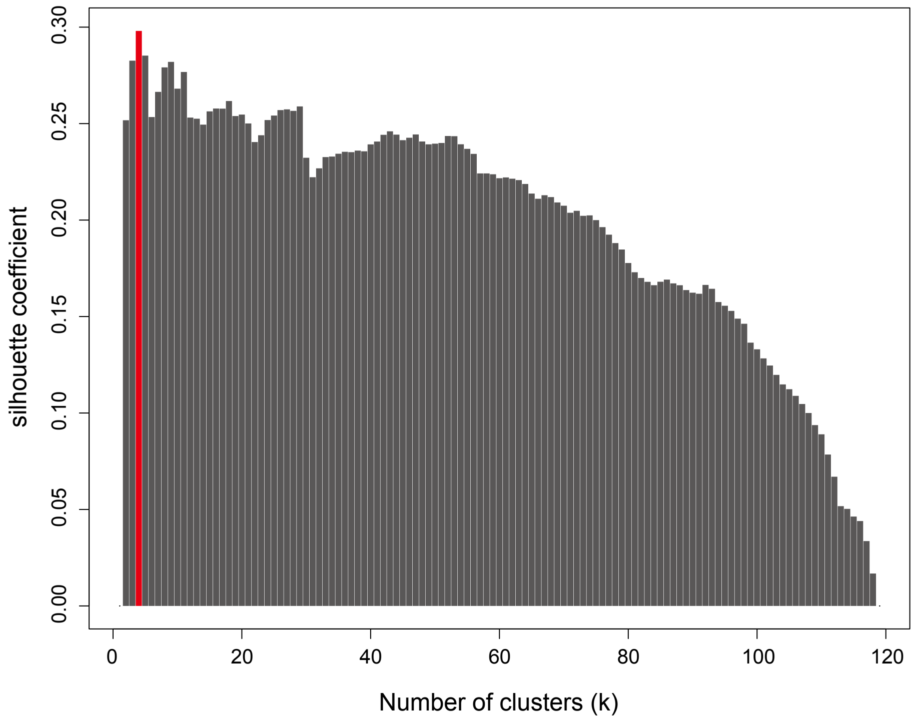


**Supplementary Figure S3. A** Cluster analysis of the amino acid compositions in low complexity regions. Green, blue, red, and orange lines indicate cluster 1, 2, 3 and 4, respectively. **B** The result of Silhouette analysis. Optimal number of clusters was estimated to four (red bar).

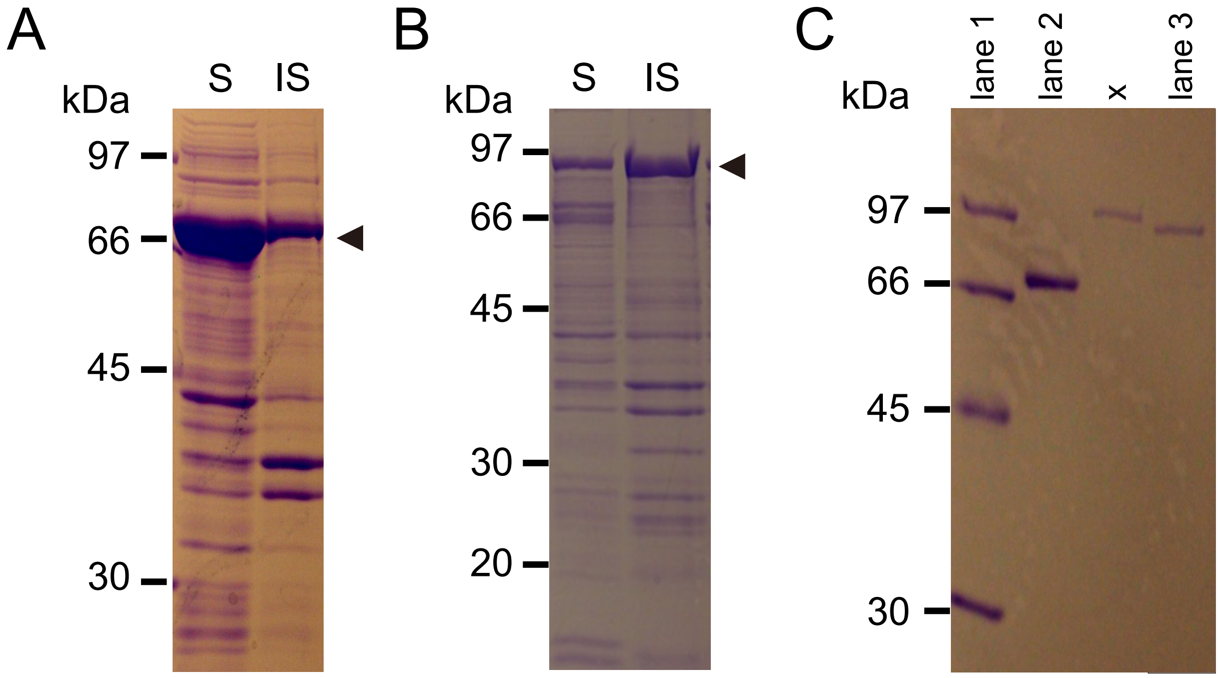


**Supplementary Figure S4.** Supplementary Figure S4. Recombinant protein expression and purification. (A) Tag-only protein (empty vector of pET44(+)) expression was induced by 1 mM of IPTG treatment at 37oC for 3h. Black arrowheads indicate target protein expression. (B) r-PfuVWA protein expression was induced by 1 mM of IPTG treatment at 20ºC for 24h. Black arrowheads indicate target protein expression. (C) Expressed proteins (Tag-only and r-PfuVWA) were purified using Ni-column. lane 1, 2, and 3 are marker, Tag-only protein, and rPfuVWA protein, respectively. S, PBS soluble fraction; IS, PBS insoluble fraction.

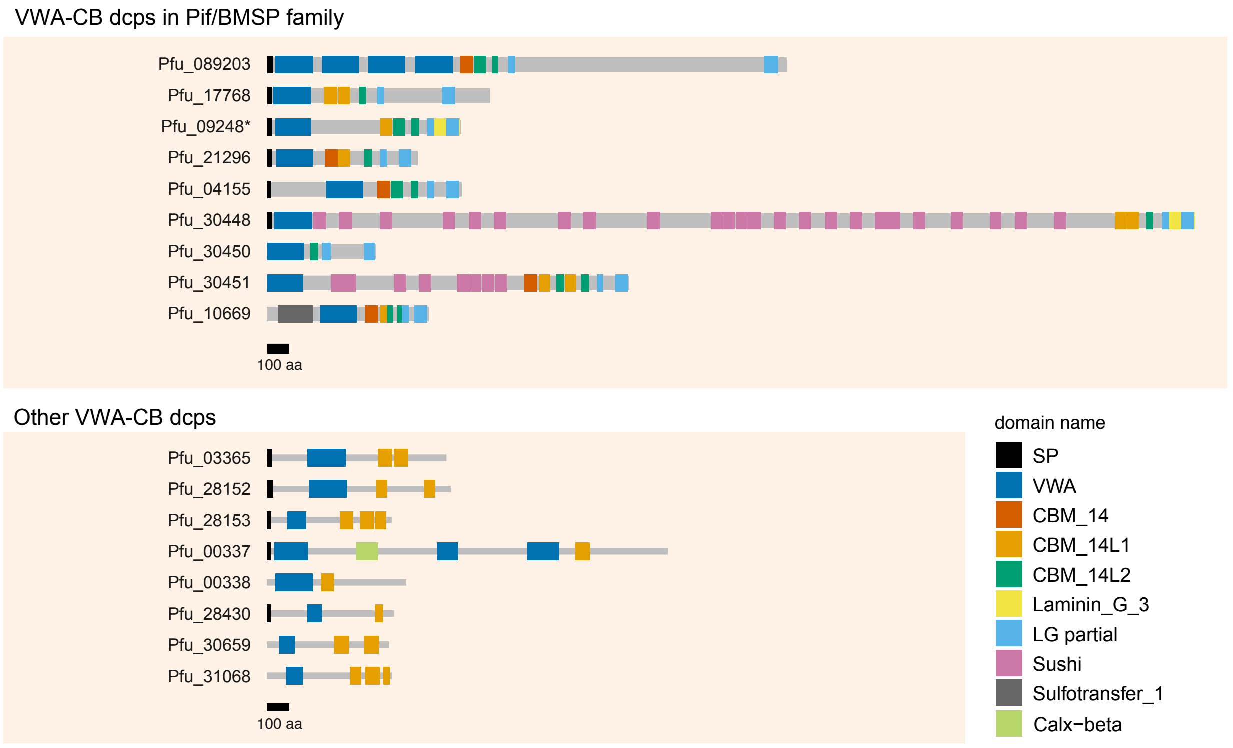


**Supplementary Figure S5.** Schematic representation of VWA-CB domains-containing proteins in *Pinctada fucata*.
